# Testing an mHealth System for Individuals With Mild to Moderate Alcohol Use Disorders: Protocol for a Type 1 Hybrid Effectiveness-Implementation Trial

**DOI:** 10.2196/31109

**Published:** 2022-02-18

**Authors:** Linda S Park, Ming-Yuan Chih, Christine Stephenson, Nicholas Schumacher, Randall Brown, David Gustafson, Bruce Barrett, Andrew Quanbeck

**Affiliations:** 1 Department of Family Medicine and Community Health School of Medicine and Public Health University of Wisconsin–Madison Madison, WI United States; 2 Department of Health and Clinical Sciences College of Health Sciences University of Kentucky Lexington, KY United States; 3 Center for Health Disparities Research School of Medicine and Public Health University of Wisconsin–Madison Madison, WI United States; 4 Department of Industrial and Systems Engineering College of Engineering University of Wisconsin–Madison Madison, WI United States

**Keywords:** mHealth, mobile health, alcohol use disorder, alcohol reduction, wellness, risky drinking, quality of life, protocol

## Abstract

**Background:**

The extent of human interaction needed to achieve effective and cost-effective use of mobile health (mHealth) apps for individuals with mild to moderate alcohol use disorder (AUD) remains largely unexamined. This study seeks to understand how varying levels of human interaction affect the ways in which an mHealth intervention for the prevention and treatment of AUDs works or does not work, for whom, and under what circumstances.

**Objective:**

The primary aim is to detect the effectiveness of an mHealth intervention by assessing differences in self-reported risky drinking patterns and quality of life between participants in three study groups (self-monitored, peer-supported, and clinically integrated). The cost-effectiveness of each approach will also be assessed.

**Methods:**

This hybrid type 1 study is an unblinded patient-level randomized clinical trial testing the effects of using an evidence-based mHealth system on participants’ drinking patterns and quality of life. There are two groups of participants for this study: individuals receiving the intervention and health care professionals practicing in the broader health care environment. The intervention is a smartphone app that encourages users to reduce their alcohol consumption within the context of integrative medicine using techniques to build healthy habits. The primary outcomes for quantitative analysis will be participant data on their risky drinking days and quality of life as well as app use from weekly and quarterly surveys. Cost measures include intervention and implementation costs. The cost per participant will be determined for each study arm, with intervention and implementation costs separated within each group. There will also be a qualitative assessment of health care professionals’ engagement with the app as well as their thoughts on participant experience with the app.

**Results:**

This protocol was approved by the Health Sciences Minimal Risk Institutional Review Board on November 18, 2019, with subsequent annual reviews. Recruitment began on March 6, 2020, but was suspended on March 13, 2020, due to the COVID-19 pandemic restrictions. Limited recruitment resumed on July 6, 2020. Trial status as of November 17, 2021, is as follows: 357 participants were enrolled in the study for a planned enrollment of 546 participants.

**Conclusions:**

The new knowledge gained from this study could have wide and lasting benefits related to the integration of mHealth systems for individuals with mild to moderate AUDs. The results of this study will guide policy makers and providers toward cost-effective ways to incorporate technology in health care and community settings.

**Trial Registration:**

ClinicalTrials.gov NCT04011644; https://clinicaltrials.gov/ct2/show/NCT04011644

**International Registered Report Identifier (IRRID):**

DERR1-10.2196/31109

## Introduction

### Overview

This paper describes the protocol for a randomized clinical trial testing an evidence-based alcohol use recovery app adapted for use as a prevention and harm-reduction app for individuals with mild to moderate alcohol use disorder (AUD). This was adapted from an app that was shown to be helpful for patients in residential treatment centers. Alcohol misuse, high-risk drinking, and AUD constitute a public health crisis in the United States [1]. This rate of high-risk drinking has substantially increased in recent years, with 1 in 8 adults reporting high-risk drinking in the past year [1]. In the study cited, high-risk drinking was defined as exceeding the recommended drinking limit of 3 in a day for women and men aged ≥65 years or 4 for men aged ≤65 years at least weekly in the past 12 months [2]. Increases in alcohol use in general and high-risk drinking predict an increase in the treatments needed for certain chronic comorbidities [3]. Improving access to effective treatment is critical for a disease as pervasive as AUD, for which few receive treatment.

### Background

The use of mobile health (mHealth) apps to improve the self-management of chronic diseases has steadily increased. A growing body of research has begun to show positive outcomes related to mHealth in the management of chronic conditions [4,5], specifically for unhealthy alcohol use [6-8].

This study uses an mHealth app called *Tula*, Sanskrit for *balance*. Tula is based on Addiction-Comprehensive Health Enhancement Support System (A-CHESS), which was one of the first mHealth apps proven effective in a randomized clinical trial of patients recovering from severe AUD [8]. In an earlier study, A-CHESS showed a 57% reduction in risky drinking days among patients using the app with standard of care compared with those who did not use an app when leaving a 90-day residential treatment for AUD. Since then, the platform has been used as an addiction recovery support and relapse prevention with thousands of patients across various settings, including at-risk veterans in upstate New York [9], women in rural Appalachia [10], and drug-court participants in Massachusetts [11]. The intervention was also adapted for specific populations; for instance, a Spanish-language version of the app was developed to test a culturally relevant recovery support service for Hispanic and Latino patients completing residential treatment in the Boston area [12]. The system has been adapted for other substance use disorders, and A-CHESS is currently being tested in a randomized trial of patients with opioid use disorder [13]. In addition, the platform—under the name *Seva*, Sanskrit for *selfless caring*—was used in the first mHealth implementation research trial that aimed to integrate behavioral health treatment into primary care [14]. The original and subsequent versions of A-CHESS have a theoretical basis in self-determination theory, which holds that helping people meet three basic needs—feeling competent, feeling related to others, and feeling internally motivated and not coerced in one’s actions—improves their adaptive functioning [15].

To our knowledge, the literature does not address the extent to which human interaction is needed to achieve the most clinically effective and cost-effective benefits of mHealth app use. Addressing these questions is essential for determining the future role of mHealth in reducing drinking and alcohol-related harm. These questions have implications for addressing substance use disorders and other chronic illnesses within health care settings.

### Objectives

This study seeks to understand how varying levels of human interaction affect the ways in which an mHealth system works or does not work, for whom, and under what circumstances. This trial tests three different mHealth support models: (1) on their own (a self-monitored, low-touch model) or in conjunction with either (2) peer support from a community organization (a medium-touch model) or (3) clinical support within a primary health care system (a high-touch model). We hypothesize that the differences in level of interaction (*human touch*) within the 3 mHealth support models will demonstrate effectiveness, allowing cost-effectiveness assessment and potential for dissemination in a population (Table 1). The low-touch model is the least costly and easiest to implement because of its low level of required support. However, if active involvement of either peer support specialists or health care staff substantially increases the effectiveness, the additional cost may be a worthwhile investment.

**Table 1:** The 3-month intervention: 3 study arms.

Self-monitored (app only)	Peer-Supported	Clinically integrated
Unguided use of Tula	Tula use supported by a community-based peer support specialist	Tula use supported by the health coach
Study team conducts safety monitoring and technical support	Interpersonal communication and wellness monitoring via the app	Up to three 1:1 health coaching sessions via phone call
No discussion forum	A discussion forum moderated by a peer support specialist	A discussion forum moderated by a health coach
No communication feature with private messaging	The private messaging feature in communication routing to a peer support specialist	The private messaging feature in communication routing to a health coach
No dashboard access	No dashboard access	Health monitoring supported by a clinician dashboard

The primary aim is to detect the effectiveness of the intervention on (1) self-reported risky drinking patterns and (2) quality of life in the three study groups (self-monitored, peer-supported, and clinically integrated). The secondary aims complement the primary aim. First, we will look at the degree to which sex (male or female) and severity of alcohol use moderates the intervention outcomes among Tula users and the degree to which patient competence, relatedness, and autonomous motivation (the 3 tenets of self-determination theory) [15] mediate the intervention effect in the Tula groups. We will also assess the cost-effectiveness of each approach. Finally, we will conduct qualitative assessment interviews to understand clinician and implementer engagement and ways to refine Tula and its associated integration for future implementation and dissemination.

### Study Design

**Figure 1:**
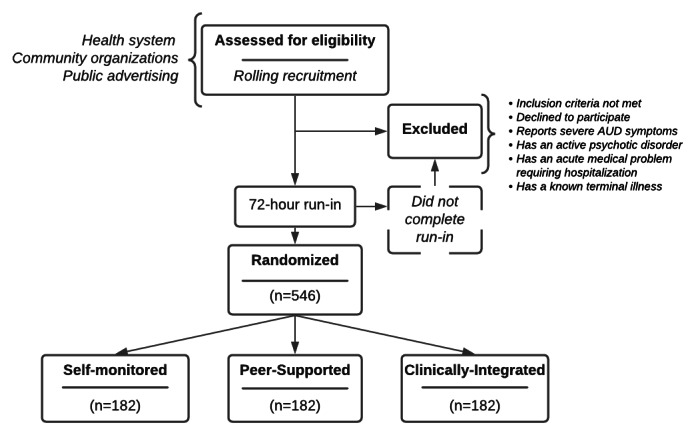
Study diagram. AUD: alcohol use disorder.

The study is an unblinded, patient-level, randomized clinical trial (Figure 1). A hybrid type I design [16] is used to test the effects of an intervention while simultaneously gathering information related to implementation. This hybrid type I trial tests the effects of the use of an evidence-based mHealth system on participants’ moderate- to high-risk drinking patterns and quality of life. The study design allows for each of the 3 randomized groups to receive an intervention variant using mHealth support models designed to support the clinical integration of behavioral intervention technologies (BITs), as described by Hermes et al [17]. The study design compares a fully automated BIT with 2 guided BITs, differentiated by graduated levels of external support (as described in detail in the Intervention section).

## Methods

### Participants, Interventions, and Outcomes

#### Study Setting

##### Overview

The study setting to recruit participants is the geographic boundaries of a large academic integrated health care system spanning several contiguous counties in a Midwestern state. The study management site is housed within the academic medical center (a university with a medical school and a teaching hospital or health care system) [18]. As an mHealth study, this study is conducted in a fully remote fashion, including partnerships with local community organizations.

##### Eligibility Criteria

There are two groups of participants for this study: (1) individuals who will receive the intervention and (2) health care professionals practicing in a broader health care environment.

##### Inclusion and Exclusion Criteria for Participants Receiving the mHealth Intervention

Participants will self-refer to the study; members of the study team will determine eligibility based on self-reported data (Textbox 1) collected via a secure, web-based screening survey.

Other substance use disorders or misuse of other substances will not preclude participants’ enrollment in the study. If the following scenarios occur during a participant’s study period, the participant will remain enrolled in the study and their circumstances will be documented by the study coordinator:

- The participant is unreachable for follow-up surveys.
- The participant becomes incarcerated. In the case of incarceration, no study data will be collected during the time they are in custody.

**Patient inclusion and exclusion criteria.**

**Inclusion criteria**

- Aged ≥21 years
- Wants to reduce drinking
- Owns a smartphone and is willing to download and use the Tula app
- Lives within the health system service area
- Meets at least one of the following criteria:
  Alcohol Use Disorders Identification Test [19] screening score ≥8 or
  Responds “yes” to at least two questions on the alcohol use disorder (AUD) or the Diagnostic and Statistical Manual of Mental Disorders-5th edition (DSM-5) [20] or
  Reports moderate- to high-risk drinking patterns (as defined by the National Institute on Alcohol Abuse and Alcoholism over the previous week):
    More than 3 drinks on any single day and more than 7 drinks per week (women)
    More than 4 drinks on any single day and more than 14 drinks per week (men)

**Exclusion criteria**

- Reports symptoms consistent with severe AUD during screening (more than 6 of 11 symptoms from DSM-5 criteria; this app does not provide support for severe AUD)
- Has an active psychotic disorder diagnosis
- Has an acute medical problem requiring immediate hospitalization
- Has a known terminal illness

##### Inclusion and Exclusion Criteria for Health Care Professionals

The selected group of health care professionals will include (1) clinicians who provided information about the study to at least one patient; (2) health care professionals who participated in interviews during the development year preceding the clinical trial; or (3) health care professionals who were involved in the implementation and could provide a health system perspective.

#### Intervention

##### Overview

Similar to previous and concurrent versions of A-CHESS, Tula operates on smartphones. Tula maintains the core components of A-CHESS but is modified to address the broad spectrum of issues related to alcohol misuse likely to be found in any community in the United States. For example, while the original version of A-CHESS [8] encouraged participants to abstain from using alcohol as a part of their AUD recovery, Tula encourages users to reduce their use of alcohol. One key adaptation is that Tula adopts the principles of the Whole Health model [18], placing alcohol use reduction within the context of integrative medicine, an adaptation made based on input from partnering health care stakeholders.

Tools and services in Tula (Textbox 2) include self-assessments; goal-setting tools and strategies; techniques for maintaining motivation and building healthy habits; information about drinking and wellness; strategies for reducing drinking and meeting other health goals; audio recordings; and other tools for stress reduction, relaxation, and personal health management.

**Tula content and tools.**

**Feature and description**

- Thought of the Day: daily inspirational quotes intended to motivate and engage participants.
- Whole Health: the Whole Health module provides information and tools to improve the whole health of a person. These topics and tools include the following: What is Whole Health; Circle of Health; Self-Care; Mindful Awareness; Whole Health Resources; and a Personal Health Inventory.
- Motivation: users can record in words and photos their reasons for wanting to work on their drinking and wellness. Other journaling and curation tools to boost motivation include “What Matters to Me,” a Gratitude journal, and “Favorites.”
- Tracker (the Tracker feature is accessed by all participants but is also monitored by the health coach in the clinically integrated group as part of the health coaching goal-setting): the Tracker tool allows users to set and review goals, track and graph their progress, and record their health and wellness patterns related to their quality of life (such as mood, sleep, social support, etc).
- Communication (communication features are limited in the self-monitored group): users can send and receive private messages with other Tula members and can access in-app discussion forums with other members of their group.
- Information: a content and resource library organized around the Circle of Health’s eight domains of self-care—Working Your Body, Sleep and Recharge, Food and Drink, Personal Development, Relationships, Mood and Mindset, Surroundings, and the Power of the Mind.
- Relaxation: information on relaxation techniques, audio recordings for guided meditation, and binaural beats.
- Strategies: tips for reducing drinking, cognitive behavioral therapy, and goal-setting.
- What are you grateful for: a daily prompt to reflect on gratitude.

##### mHealth Implementation Models

Table 1 outlines the key characteristics of the study arms during the 3-month active intervention period for each patient. All participants had access to the same basic content in Tula. The key differences relate to the level of human touch available by study group assignment.

##### Self-monitored Group (Low Touch)

Participants will use the app on their own, just as they would any commercially available health app downloaded to their smartphone. There is no access to a discussion forum and private messaging, or the aid of an external care team or social support. Participants may reach out to the study team by email, phone, or from within the app via the “Messages from the Researchers” tool. Communication is only intended for participants to ask questions about the app, about the study, and receive technological support. The study team conducts routine safety monitoring based on participant use data and any communication initiated by the participants to the study team.

##### Peer-Supported Group (Medium Touch)

Participants have access to social support and access to certified peer support specialists. Peer support specialists are staff members from a community partner outside of the health care system. Newly randomized participants receive a welcome message via the private messaging feature in Tula from peer support specialists. To maintain anonymity, all participants identify themselves using their username. The participants’ main interactions are with other members in the same group through discussion forums where they share posts. Peer support specialists moderate and participate in the discussion forums while encouraging the use of Tula (eg, by posting topics in the discussion forum, pointing Tula users to a potentially helpful tip). They have no access to study data and only know the participants through their usernames.

##### Clinically Integrated Group (High Touch)

Participants have access to a discussion forum specific to this group and have access to a certified health coach. Health coaches, as employees of the health care system, help individuals make lifestyle changes to achieve their goals for health and wellness. Participants can have 3 one-on-one personal health coaching sessions (via phone) during the active 90-day implementation period. Health coaches will provide a structure for participants to achieve their self-identified goals by helping them envision a healthier lifestyle as they reduce their alcohol consumption. Participants can opt to share selected Tula data with the health coach through a dashboard—data on drinking days or drinks per day and other data reported via weekly surveys. These data allow health coaches to provide more responsive support to participants in meeting their goals for alcohol use.

#### Outcomes

##### Overview

Table 2 shows the outcomes and the mediators and their measurement. The definition and psychometric properties of each measure are also listed.

**Table 2:** Outcomes and measures.

Dimension			Measure		Source		Timing (after randomization)
**Primary outcomes**							
	Risky drinking days	Timeline follow back [21,22]		Participant survey		0, 3, 6, 9, and 12 months	
	Quality of life	PROMIS^a^ Global Health [23] and 2 COVID-19 impact items		Participant survey		0, 3, 6, 9, and 12 months	
**Cost outcomes**							
	Health care use	Medical services utilization form [24]		Participant survey		0, 6, and 12 months	
	Implementation costs	COINS^b^ [25]		Health care professional interviews		Every 6 months	
**Mediators**							
	Relatedness	McTavish Bonding Scale [26]		Participant survey		0, 3, 6, 9, and 12 months	
	Competence	Perceived Competence Scale		Participant survey		0, 3, 6, 9, and 12 months	
	Autonomous motivation	TSRQ^c^		Participant survey		0, 3, 6, 9, and 12 months	
	Tula use (patients)	Number of days used; number of pages viewed		Server log files		Continuous	
**Other outcomes**							
	Risk and protection factors	Brief Alcohol Monitor (revised) [27]		Participant survey		Weekly	
	Tula use (clinicians)	Number of days used		Server log files		Continuous	
	Patient characteristics	Race, ethnicity, biological sex, and age		Participant survey		0 months	

^a^PROMIS: Patient-Reported Outcomes Measurement Information System.

^b^COINS: Cost of Implementing New Strategies.

^c^TSRQ: Treatment Self-Regulation Questionnaire.

##### Primary Outcomes

The primary outcomes include patient-reported risky drinking days and quality of life. Risky drinking days are the number of days on which a participant’s drinking in a 2-hour period exceeded 4 standard drinks for men and 3 standard drinks for women, defined using the National Institute on Alcohol Abuse and Alcoholism’s definition of a standard drink as 1 containing 14 g of 1 alcohol (12 oz of regular beer, 5 oz of wine, or 1.5 oz of distilled spirits) [2]. Risky drinking days are measured using the patient-reported timeline follow back survey. The timeline follow back survey consistently demonstrates reliability for assessments in recall periods as long as 6 months, test-retest reliability of ≥0.80, and convergent and discriminant validity with other measures; it also correlates with collateral reports and urine toxicology tests [28]. Quality of life will be measured using the Patient-Reported Outcomes Measurement Information System (PROMIS) Global Health scale, a 10-item subjective measure of general health [29]. It includes a 4-item global physical health scale (Cronbach *α*=.81), a 4-item global mental health scale (Cronbach *α*=.86), and two additional items—general health and satisfaction with social roles—that can each be scored as a single item. The PROMIS scales were developed using item response theory and capture a greater range of the trait being measured with greater precision than other instruments. At the beginning of the COVID-19 pandemic, we added two 5-point Likert scale items asking about the impact of the COVID-19 pandemic on participants’ physical and mental health.

##### Cost Outcomes

Cost measures include health care use costs and intervention and implementation costs. Health care use will be collected from the self-reported medical services utilization form [24] (6 items). Intervention costs will be determined using Tula time-stamped log files and billing codes logged by the health coach. Implementation costs will be determined through clinic staff interviews at the end of the intervention period using the Cost of Implementing New Strategies model [30]. The cost per participant will be determined for each study arm (self-monitored, peer-supported, and clinically integrated) with intervention and implementation costs separated within each group.

##### Mediators

Several mediators include the 3 fundamental psychological needs based on the self-determination theory [15] and the Tula use data. Among the 3 core needs specified by self-determination theory, relatedness will be measured using the McTavish 5-item Bonding scale [26], which is highly correlated with other social support scales, has a high reliability of 0.9, and has been found to mediate effects between patients’ use of eHealth systems and their coping behaviors [26,31]. Competence will be measured by the perceived competence scale, a 4-item alcohol-addiction focused scale rating confidence in the ability to cope in high-risk situations; reported reliability was above 0.9 [32]. Autonomous motivation will be measured by the modified Treatment Self-Regulation Questionnaire, which uses a 7-point Likert scale to respond to 5 items assessing the degree to which a person’s motivation for healthy behavior is autonomous. Previous tests found good reliability (Cronbach *α*=.88) and predicted changes in health-related behavior (*P*<.001). Participant use of Tula will be measured by the number of days using Tula and the number of pages viewed. Days of use and pages were found to be associated with a reduction in risky drinking days among AUD participants [8]. Clinician use of Tula will be measured by counting the number of days per month in which clinicians log into the system during the intervention period and the pages viewed.

#### Participant Timeline

**Figure 2:**
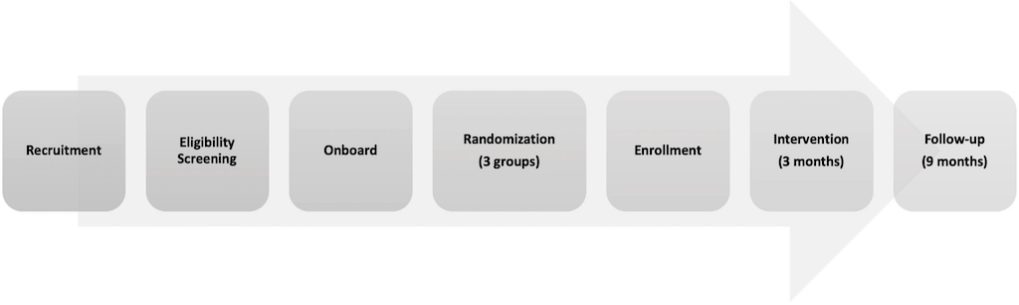
Participant timeline.

Participants’ involvement in the study can be divided into four distinct phases: (1) a pre-enrollment period encompassing screening completion, evaluation by the study team, and downloading the app; (2) an enrollment period consisting of a 2-stage consent process framing a 72-hour run-in period, a phone call with a member of the research team, randomization, and the final confirmation of enrollment; (3) a 3-month active intervention period; and (4) a 9-month follow-up period (Figure 2).

The pre-enrollment and enrollment phases, together, may range from approximately 48 hours to 3 weeks, depending on the participant’s responsiveness and availability. Once enrolled, a participant will be engaged in the study for 12 months.

### Sample Size

This study was designed to detect the differences in the two primary outcomes, Risky Drinking Days and Quality of Life, among the 3 groups. In a previous study [33], after a 6-month web-based intervention study, alcohol users in the internet-based, therapist-led group reported having fewer drinks (Cohen *d*=0.38) and a better quality of life (Cohen *d*=0.44) than those in the internet-based, self-help group. In this protocol, sufficient power (1-β=.80, multiple comparison adjusted Cronbach *α*=.00833, 2-tailed) to detect a more conservative effect size of Cohen *d*=0.25 in a design with 4 repeated measurements with a first-order autoregressive covariance structure (correlation *ρ*=0.3) would require approximately 182 participants per group (or a total of 546), assuming 28% attrition. Using prior research [14] to estimate the SD of risky drinking days, the effect of Cohen *d*=0.25 would equate to a difference of approximately 0.24 risky drinking days per week and 1.62 overall quality of life value measured by the PROMIS Global Health instrument.

### Recruitment

#### Participant Recruitment

Recruitment for this study relies on raising its visibility among key groups of stakeholders and tailoring our outreach efforts to be relevant and sensitive to each group’s needs and interests. The study’s recruitment strategy has centered on three key areas: clinical settings, community-based organizations, and the public media marketplace.

By promoting the study to health care professionals, primary care providers, social workers, emergency departments, and behavioral health specialists operating within the local health systems, we enlisted the help of clinical study champions and aim to provide information and resources they can share with patients who may be eligible for the study. Engaging with local leaders from underrepresented and marginalized communities and working to build or fortify relationships with community-based organizations and partners is critical for promoting the study in a way that invites participation and inclusion of diverse voices, perspectives, and experiences. Finally, the use of targeted digital and print media enables the study team to promote the study broadly but strategically while providing information and materials that enable potential participants to learn about the study in a way that maintains discretion, privacy, and autonomy.

All participants who complete the eligibility screen receive a US $10 incentive, delivered in the form of a digital gift card when the study team notifies them of their eligibility.

#### Recruitment of Health Care Professionals (After the Intervention)

Upon completion of the study’s active intervention and follow-up period, the study team will invite a select group of health care professionals to participate in the interviews. Email invitations will ask if they would like to share their experiences. Details of the study and their participation will be explained, and if they agree to participate in the study, signed informed consent will be collected in person, at the time of the interview, by a member of the study team.

### Assignment of Interventions

#### Randomization

The randomization list was generated using the block randomization procedure in the Power Analysis and Statistical Software (PASS 2020) and stratified for participant-reported biological sex (male or female) and alcohol use severity (mild or moderate) based on Diagnostic and Statistical Manual of Mental Disorders, fifth edition (DSM-5) scores (eg, mild=DSM-5 score 2-3 and moderate=DSM-5 score 4-6) calculated from participants’ responses in the pretest survey (Table 2).

To prepare the randomization tool, the program manager of the study team entered the sequence of randomization assignments for each stratification group into a protected spreadsheet and masked the data. During randomization, a researcher from the study team conducts an active call with the participant, enters the participant’s study ID into the next available placement in the randomization sequence, and reveals or unmasks the participant’s group assignment. Although on call with the participant, the researcher configures the participant’s permissions in the app based on their group assignment and confirms that the participant has access to the version of the app that corresponds to their placement.

Before the randomization call, to limit or avoid interaction between the participants and the study team members responsible for conducting randomization, each stage of the pre-enrollment and enrollment process (screening, eligibility communication, scheduling, and randomization) is led by a different member of the team.

#### Enrollment

Potential participants will complete a web-based eligibility survey on the research team’s website. Eligible participants will receive an invitation to participate with instructions to begin the enrollment process and a link to download the app. They may receive up to 2 email reminders during the 2 weeks following their completed screening.

When participants opened the app, the first of 2 digital informed consent documents appeared. Participants may contact the study team by email or phone with questions or concerns before electronically signing the consent form. If a participant declines to participate (eg, do not consent), reasons for refusal (if provided) will be documented, in keeping with CONSORT (Consolidated Standards for Reporting Trials) standards [32]. The study team will not initiate further contact with the participant.

Once participants consent and submit the digital consent form, they can create a user account initiating a 72-hour run-in period where they complete the baseline survey bundle (10 surveys, 64 questions in total). Participants who do not complete the 72-hour run-in are not randomized; their account is deactivated; they are removed from the study; and the reason for exclusion (“did not complete run-in”) is documented accordingly.

Participants completing the 72-hour run-in are invited to schedule a call for the final step in the enrollment process. During the call, a member of the study team reviews the study and first consent form, answers any questions, and completes the randomization process. Once the app permissions have been reconfigured to the appropriate study group, the participant receives a second informed consent to review and sign. The second consent document describes the study activities specific to the participant’s group assignment. Both informed consents are stored in the Study Information page in Tula for the participants’ records with an option to print a paper copy.

Participants may withdraw their consent at any time throughout the 12-month period after their enrollment, at which time the study team will document their request, deactivate their account, and send a final confirmation. The participant will not receive further communication from the study.

### Data Collection, Management, and Analysis

#### Quantitative Data Collection

The data collected by the Tula system come from multiple sources (Table 2). All surveys are conducted in the Tula app. Participants receive survey notifications from the Tula app on their phones and then complete the survey in the Tula app accordingly.

#### Baseline and Quarterly Surveys

##### Weekly Surveys

Tula prompts participants to take weekly surveys administered via the app to track their drinking and quality of life. Participants report their drinking using the 7-day timeline follow back [21,22] survey. A modified version of the Brief Alcohol Monitor [27] survey (10 items) allows participants to track individual risk and protection factors that may influence problematic alcohol use.

##### Tula Use

Tula use data are collected in time-stamped log files and include when a participant accessed Tula, the services used, duration of service use, pages viewed, messages posted and received, and content of messages. Tula use measures the dose of the intervention received for dose and response analyses.

##### Health Care Use

Health care use will be collected from the Self-reported Medical Services Utilization Form 61 (6 items; see Table 2 for frequency).

##### Intervention and Implementation Costs

Intervention costs will be determined using Tula time-stamped log files and billing codes logged by the health coach and peer mentors. Implementation costs will be estimated through health care professional interviews at the end of the intervention period and organized according to the Cost of Implementing New Strategies model [25]. The cost per participant will be determined for each study arm (self-monitored, peer-supported, and clinically integrated) with intervention and implementation costs separated within each group.

#### Qualitative Data Collection

Interviews will take place once the intervention phase of the study is complete and last 30-60 minutes. Open-ended questions will allow health care professionals to assess their experiences with Tula and provide feedback on implementation as well as their thoughts on participants’ experiences using Tula. Interviews will be conducted in a private location convenient for participants, either in clinician offices or via a secure videoconference call. All interviews will be audio-recorded and transcribed.

#### Retention or Adherence

Participants who complete their enrollment in the study are eligible for renumeration. Study incentives are built into the first 12 weekly surveys and 4 quarterly follow-up surveys at months 3, 6, 9, and 12. How to earn the incentives (gift cards) and how the incentives will be distributed monthly to participants by digital gift code sent via the message feature in Tula are explained to participants during the final step of enrollment. At the end of the 12-month study period, participants could potentially earn up to US $250 in gift cards.

#### Data Analysis

##### Primary Analysis

The analysis assesses the direct treatment effects on participant outcomes over time. We will construct a longitudinal model of the outcome measures at 3, 6, 9, and 12 months after randomization. Variables, stratified by sex and severity of alcohol use, will be included as factors in the model. The baseline values of the outcome will be included as covariates, with a separate model for each primary outcome (risky drinking days and quality of life). This longitudinal analysis is complicated by the dependence on successive observations made on the same individual. Furthermore, as complete control of measurement is not possible, there may be incomplete data from individual participants. Therefore, we will conduct a mixed-model analysis of repeated measures based on the general linear model with the assessment of various covariance structures (compound symmetrical, autoregressive order one, and unstructured). Covariance structure selection is based on the Akaike information criterion and Schwarz Bayesian criterion [23,24]. Pairwise comparisons between treatment groups and specific treatment time contrasts in the mixed model will be conducted to respond to between-group effects and time-based effects.

##### Descriptive Analyses

The research team will use descriptive statistics for all demographic and clinical variables across all 3 arms. To assess the impact of chance baseline imbalances between arms on intervention effect estimates, variables with noticeable differences will be included as covariates in a sensitivity analysis.

##### Mediation and Moderation Analysis

To augment the intervention analysis, we will estimate the direct and indirect effects that groups have on the outcomes by mediating variables using the structural equation model method. To test moderation effects, the interaction of the moderators (gender and severity of alcohol use severity) and randomization will be added to the model and estimated separately. We will examine the magnitude and direction of differential intervention effects between levels in these moderators (eg, male vs female).

##### Cost-effectiveness Analysis

Operational cost will be calculated based on the tenets of engineering economics. This study will use incremental cost-effectiveness ratios to compare the clinically integrated, peer-supported, and self-monitored groups. The incremental cost associated with reductions in drinking days and improvements in quality of life will be calculated over the 12-month intervention period.

##### Qualitative Data Analysis

Content analysis will describe how Tula use can improve patient outcomes, identify potential improvements in Tula, and identify how qualitative data can supplement the quantitative analysis. A qualitative researcher will construct a coding scheme to assess the ideas of the study to capture references to a concept. The analyses will help the research team refine Tula for future dissemination by determining the individual and organizational conditions necessary to promote effectiveness.

### Data Monitoring

All study data collected by the app will be stored in secure password-protected servers. No patient health information will be collected from their electronic medical records, and no data collected by the app will be entered into patients’ electronic medical records or affect the legal medical record. All participants are assigned an ID number, and all data will be deidentified before exporting for statistical analyses. Any hard copy–identifying information will be stored in a locked cabinet. When all study activities are complete, audio recordings, participant ID, and other identifiable information will be destroyed; only the deidentified code will remain.

With consultation from the funding agency (National Institute on Alcoholism and Alcohol Abuse) and the institutional review board, this protocol was exempted from requiring a formal data monitoring committee review.

### Ethics Approval

This protocol was initially approved by the Health Sciences Minimal Risk Institutional Review Board (2019-0337) with subsequent annual reviews.

## Results

This study is currently ongoing. Recruitment began on March 6, 2020, but was suspended on March 13, 2020, due to the COVID-19 pandemic restrictions. Limited recruitment resumed on July 6, 2020. The trial status as of November 17, 2021, is as follows: 357 participants have been enrolled in the study toward a planned enrollment of 546 participants. This study is expected to conclude on September 1st, 2023.

## Discussion

### Challenges

With the ubiquity of smartphones, the use of mHealth apps to improve the management of chronic diseases, including unhealthy alcohol use, is increasing. However, the extent of human interaction needed to achieve effective and cost-effective benefits of mHealth remains a challenge. The study is designed to detect differences in the costs and effectiveness of implementing an mHealth intervention using 3 strategies that systematically vary the level of human touch provided to support its use by patients. Such a novel approach to implementing an mHealth system presents several challenges, primarily stemming from disruptions associated with the COVID-19 pandemic. One of the biggest challenges involves outreach and recruitment in communities of color. Creating trust in messaging, which was already a challenge, was doubly impacted by the COVID-19 pandemic as well as by police brutality in African American communities nationwide. Although an mHealth study by design is digital and remote, more challenges arose regarding ways to engage with clinics and community organizations for recruitment, particularly with local leaders from underrepresented and marginalized communities. Finally, with increased reliance on digital technologies caused by the pandemic, there was an unexpected surge in internet bot activities, creating a spike in false screening results that required increased safeguarding and monitoring by the research team.

### Lessons Learned

There have been lessons learned, aside from the challenges encountered in executing this protocol. Getting clinician feedback and conducting usability tests of the app with potential participants before recruitment helped create a broader and more user-friendly mHealth intervention and implementation climate. Developing a flexible and adaptive recruitment strategy was also an important lesson learned for a study reliant on participant self-referral, especially when encountering unexpected situations such as the novel COVID-19 pandemic. Another lesson learned has been understanding the varied forms that the stigma of alcohol use presents itself within different communities and cultures. With alcohol misuse, high-risk drinking, and AUD constituting a public health crisis in the United States, testing whether an app such as Tula can benefit patients in a primary health care system is essential in determining the future role of mHealth in reducing drinking and alcohol-related harm. The results of this study may also provide guidance to policy makers and health care decision makers on the most cost-effective ways to incorporate technology in health care settings.

## Abbreviations

A-CHESS: Addiction-Comprehensive Health Enhancement Support System
AUD: alcohol use disorder
BIT: behavioral intervention technology
CONSORT: Consolidated Standards for Reporting Trials
DSM-5: Diagnostic and Statistical Manual of Mental Disorders, fifth edition
mHealth: mobile health
PROMIS: Patient-reported Outcomes Measurement Information System
SPIRIT: Standard Protocol Items: Recommendations for Interventional Trials
